# Associations Between Behavioral Effects of Bisphenol A and DNA Methylation in Zebrafish Embryos

**DOI:** 10.3389/fgene.2019.00184

**Published:** 2019-03-08

**Authors:** Pål A. Olsvik, Paul Whatmore, Sam J. Penglase, Kaja H. Skjærven, Marc Anglès d’Auriac, Ståle Ellingsen

**Affiliations:** ^1^Institute of Marine Research, Bergen, Norway; ^2^Norwegian Institute for Water Research, Oslo, Norway

**Keywords:** zebrafish embryos, bisphenol A, behavior, gene expression, DNA methylation, epigenetics

## Abstract

Endocrine-disrupting contaminants have been associated with aberrant changes in epigenetic pathways in animals. In this study, zebrafish embryos were exposed bisphenol A (BPA) to search for associations between behavior and epigenetic mechanisms in fish. For concentration-dependent responses, embryos were exposed to a range of BPA concentrations (0.1 nM to 30 μM). Embryos were analyzed for locomotor activity at 3-, 4-, and 5-days post fertilization (dpf) in response to changing light conditions. Based on concentration-dependent effects on behavior and gene expression, 10 μM BPA [from 24 to 96 hours post fertilization (hpf)] was used for a whole-genome bisulfite sequencing (WGBS) study searching for genome-wide impacts on DNA methylation. Over the examined concentration ranges, hyperactivity was demonstrated for exposures to 0.001 μM BPA in comparison to embryos exposed to lower or higher BPA concentrations. Transcriptional analysis showed significant effects at >0.01 μM BPA for two genes related to DNA methylation (*dnmt1*, *cbs*). BPA exposure did not significantly affect global DNA methylation, but 20,474 differentially methylated (DM) sites in 4,873 genes were identified by WGBS analysis. Most DM sites were identified within gene bodies. The genes with the most DM sites were all protocadherin 2 gamma subfamily genes, related to axon targeting, synaptic development and neuronal survival. KEGG pathways most significantly affected by BPA exposure were phosphatidylinositol signaling system, followed by VEGF and MAPK signaling pathways. This study shows that BPA can affect zebrafish embryo swimming activity at very low concentrations as well as affecting numerous methylated sites in genes which are overrepresented in functionally relevant metabolic pathways. In conclusion, altered methylation patterns of genes associated with nervous system development might lead to abnormal swimming activity.

## Introduction

As part of an ongoing effort to study the mechanistic effects of endocrine-disrupting contaminants found in Atlantic salmon (*Salmo salar*) feeds, we are using zebrafish (*Danio rerio*) embryos and bisphenol A (BPA) as a model to study behavioral and molecular endpoints of chemical exposure in farmed fish. Aquaculture feeds may contain significant concentrations of contaminants such as dioxins (PCDD/PCDF), PCB and PBDE, and pesticides such as DDE and chlorpyrifos (Berntssen et al., 2010, 2011; Nacher-Mestre et al., 2014). Most of these contaminants will bioaccumulate in fish.

Many studies have described associations between endocrine-disrupting contaminants and the epigenome in fish (Iida et al., 2006; Aniagu et al., 2008; Mirbahai et al., 2011; Olsvik et al., 2014). BPA, one of the most ubiquitous plasticizing compounds and best-studied endocrine disruptors (Cole et al., 2011; Rubin, 2011; Rochester, 2013), has been associated with altered DNA methylation in mammalian cells (Walker and Gore, 2011). In fish, BPA, acting on DNA methylation, affects numerous mechanisms; immune system (Lam et al., 2011), reproductive function (Laing et al., 2016; Chen et al., 2017), antioxidant enzyme activity (Wu et al., 2011), detoxification (Lindholst et al., 2003; Bonefeld-Jorgensen et al., 2007; Olsvik et al., 2009; Maradonna et al., 2014; Bugel et al., 2016), and larval and adult behavior (Saili et al., 2012, 2013; Wang et al., 2013; Little and Seebacher, 2015; Li et al., 2017). Hyperactivity has been reported in zebrafish larvae exposed to ≥10 nM BPA (Saili et al., 2012). Significantly decreased swimming speed in response to alternating light stimulation has been observed in 120 hpf zebrafish larvae exposed to 1–15 μM BPA from 6 to 96 hpf (Wang et al., 2013).

In developing zebrafish, it has been shown that transient exposure to BPA results in hyperactivity (Saili et al., 2012), a response associated with altered gene expression (Saili et al., 2013). At 0.1 μM BPA exposure, suppressed expression of genes involved in nervous system development and function has been associated with altered behavioral responses (hyperactivity) to light stimuli in zebrafish embryos (Saili et al., 2013). Reduced expression of DNA methyltransferase 1 (*dnmt1*), as well as reduced levels of global DNA methylation, has been observed in tissues of zebrafish exposed to environmentally relevant concentrations of BPA (Laing et al., 2016). Although BPA is known to impact behavior, gene expression and DNA methylation in developing zebrafish larvae, more knowledge is needed on concentration-dependent responses and genome-wide DNA methylation to better understand the epigenetic impact of such chemicals. Conservation of DNA methylation patterns has been observed between various animals (Feng et al., 2010) and we postulate that the underlying molecular mechanisms imposed by BPA and other endocrine-disrupting chemicals will offer sufficient overlap to make comparative studies valuable.

The aim of this study was to attempt to link behavioral responses to changes in global DNA methylation in fish exposed to BPA, selected as a model endocrine-disrupting chemical. In order to determine the lowest BPA concentration affecting behavioral endpoints, zebrafish embryos were exposed to BPA concentrations between 0.1 nM and 30 μM (22 ng/L – 6.8 mg/L) for 24 h during a critical period of embryonic development (2–26 hpf). A set of transcriptional markers were also quantified to assess concentration-dependent effects of BPA in the embryos. To improve the chances of finding significant DNA methylation effects we selected a dose of 10 μM for the 72-h exposure experiment. This dose was selected based on the Saili et al. (2012, 2013) studies and our own preliminary study that all showed no lethality below 30 μM BPA, and locomotor analyses which showed linear significant responses to BPA exposure at concentrations between 1 and 30 μM BPA. This treatment (10 μM BPA) was used to identify methylated genomic regions with whole-genome bisulfite sequencing (WGBS) through later stages of embryonic development (24–96 hpf), and to elucidate possible links between behavioral, transcriptional and methylation endpoints. Other studies have examined DNA methylation in zebrafish as a response to BPA exposure, but they examined only a handful of promoters and used generalized quantification methods such as luminometric-based assay (LUMA) to assess global DNA methylation. WGBS allows a much more comprehensive analysis by examining individual methylated (and, importantly, unmethylated) cytosines across the entire genome. To our knowledge, this is the first study to use WGBS in search of BPA-induced effects on genome-wide DNA methylation in zebrafish embryos.

## Materials and Methods

### Embryo Exposure to BPA

Fish were maintained and experiments conducted in compliance with the Norwegian Animal Welfare Act guidelines. Zebrafish embryos for the exposure experiments were obtained from wild type brood stock (AB ♀ and TL ♂ strains). These adult fish were maintained under standard conditions (28.5°C, 500 μS EC, pH 7.6, 10% daily water exchange, 14:10 light:dark photoperiod) in a closed recirculation system (Aquatic habitats, Apopka, FL, United States). The adults were fed twice daily to satiation with a mixture of first instar *Artemia* nauplii (Sanders, South Ogden, UT, United States) and Gemma Micro 500 (Skretting c/o Trouw France S.A., Vervins, France). Prior to mating, adult fish were removed from the recirculation system and separated by sex within the 9 L spawning tanks (Techniplast, Buguggiate, Italy) in system water at sex ratios averaging 1♂:2♀, in the afternoon prior to mating. On the morning of the next day, the two sexes were combined in a spawning tank containing fresh system water, which was placed on a ≈20° degree lengthwise slope to enhance mating success. The fish were then allowed to interact for a 1–1.5 h period before spawned eggs were collected.

Spawned eggs were pooled, rinsed with and then placed into E3 embryo medium and sorted into ≈70 eggs/well in six well plates (Nunclon Delta Surface, Thermo Scientific, Roskilde, Denmark). Our aim was to expose embryos to sublethal graded concentrations of BPA (≥99% purity, Sigma-Aldrich, MO, United States) from 2 to 26 hpf (24 h exposure during critical development period) for behavioral and transcriptional analysis, and from 24 to 96 hpf (72 h exposure) for methylation analysis. BPA was dissolved in dimethyl sulfoxide (DMSO, >99.9% purity, Sigma-Aldrich) to make a stock solution (100 μM BPA in DMSO). Between 2 and 2.5 hpf, excess E3 embryo medium was removed from the center bottom of each well, allowing eggs to gather in the depression present at the edge. Four milliliters of treatment medium was then added to each well (three wells per treatment). Treatments were E3 media with 0.1% DMSO and one level of 0, 0.0001, 0.001, 0.01, 0.1, 1, 10, 30 μM of BPA. Embryos used to identify methylated genomic regions were only exposed to 10 μM BPA. Higher levels were not used as they result in high levels of embryo deformity (90 μM BPA; 100% deformity at 24 hpf, personal observation). In addition, there was a control (E3 media) without DMSO or BPA.

Eggs were then maintained in an incubator at 28.5°C with a light:dark cycle of 16:8 h for the trial period. We exposed embryos from 2 to 26 hpf. At 4–6 hpf, ≈50 embryos from each well were transferred to a new 6-well plate, the excess media removed from the well as before, and the treatment continued by adding another 4 ml of the respective treatment solutions. At 26 hpf (24 h of exposure), treatment solutions were removed from wells and rinsed twice for 5 min using E3 media. Each replicate of embryos was then transferred to the well of a 6-well plate, the remaining media removed and replaced with E3 (≈8 ml). Thereafter, E3 media was replaced (≈80% exchange) daily.

### Embryo Analysis – Survival, Hatch, and Deformities

Embryo mortality was measured at 24, 48 (one otolith length migration away from the eye), 72, 96, and 120 hpf, and deformities were evaluated at 24, 48, and 72 hpf. Deformities were evaluated by scanning embryos at 10× magnification, with further evaluation of those embryos found to be deformed at 20× magnification, using a stereomicroscope. Hatching percentage was calculated at 48, 54, 72, and 96 hpf.

### Larval Locomotor Activity

At 72 hpf, eight embryos showing no signs of deformities from each treatment replicate (24 embryos/treatment) were used for locomotor analysis. Larvae were transferred to individual wells of a 96-well plate in a randomized design, and analyzed for locomotor activity at 3, 4, and 5 dpf in response to changing light conditions, using an automated video tracking system (Zebralab, Viewpoint, Lyon, France) in a similar manner as previously described (Penglase et al., 2014). Several methods of locomotor analysis were conducted in this trial, both to assess the effects of BPA on larval locomotor activity, and as part of a larger project to refine methods for effectively analyzing larval locomotor activity.

*Locomotor analysis variant 1*: The method for this variant was similar to that previously described (Burgess and Granato, 2007). Modifications to this method were the threshold levels for locomotor activity (Low:Medium:High activity, 7:15:50), the length of analysis (5 min light followed by 5 or 10 min dark, data binned into 5 min lots). With these settings, low activity was where no movement occurred, so data were further grouped into either no (low) or active (medium + high) locomotor activity for statistical analyses. The same plate was used on each day, and maintained in the incubator when not being analyzed for locomotor activity. A total of eight larvae were utilized per replicate, except for the DMSO control (16 larvae) and the E3 control (24 larvae). Larvae were analyzed with this method at 3, 4, and 5 dpf. *Locomotor analysis variant 2*: This method was similar to variant one, but aimed to determine the effects of BPA on the response to dark “flashes” (short periods of darkness). The response of zebrafish larvae to dark flashes is described by Burgess and Granato (2007). Differences from variant one were the length of analysis (1 s dark followed by 9 s light × 3, total analysis length 30 s), binning (5 s) and larval age (this method was only utilized at 4 dpf). *Locomotor analysis variant 3*: This method was similar to variant two, with differences being length of analysis (0.5 s dark followed by 2.5 s light × 3, total analysis length 9 s), binning (1 s) and larval age (this method utilized at 4 and 5 dpf). *Locomotor analysis variant 4*: This method aimed to analysis larval locomotor activity under less confined conditions. In this method, two larvae from each replicate (six per treatment) were placed into individual wells of 12 well plates and wells were adjusted to ≈1 ml of E3. Larval locomotor activity was then analyzed in the viewpoint system with the tracking mode (5 min light followed by 5 min dark, data binned into 5 min lots). In addition to variants 1–3, this method enabled the analysis of the distances moved by the larvae when moving slowly (between 1 and 3 mm/s) or quickly (>3 mm/s). Other settings within the viewpoint system utilized for this analysis were “transparent” larvae, and sensitivity of 108. Due to the limited number of wells on a 12-well plate, only the DMSO control and 1, 10, and 30 μM exposure groups were analyzed with this method. Larvae were analyzed with this method at 5 dpf.

### Sampling

At 5 dpf, embryos from the locomotor activity trial were pooled with the remaining embryos from that replicate and the embryos euthanized (≈0.4 g/L MS222 neutralized with 0.4 g/L NaHCO_3_). The embryos (30–50 depending on treatment) were then transferred to Eppendorf tubes, the remaining E3 solution removed (100 μL remaining), and 0.3 mL of Trizol reagent (Invitrogen, Life Technologies, Carlsbad, CA, United States) was added. Samples were incubated at room temperature for 5 min, and homogenized by hand shaking for 1 min and then frozen and stored at -80°C until analysis.

### RNA Isolation and RT-qPCR

Zebrafish embryos (pools of 40 per sample) were homogenized using ceramic beads CK28 and a Precellys 24 homogenizer (Bertin Technologies, Montigny-le-Bretonneux, France). Total RNA was extracted using RNA Tissue Mini Kit (Qiagen, Hilden, Germany) and the BioRobot EZ1, treated with DNase according to the manufacturer’s instructions and eluted in 50 μL RNase-free MilliQ H_2_O. Quality and integrity of extracted RNA were validated with the NanoDrop ND-1000 Spectrophotometer (NanoDrop Technologies, Wilmington, DE, United States) and the Agilent 2100 Bioanalyzer (Agilent Technologies, Palo Alto, CA, United States).

PCR primer sequences used for quantification of the transcriptional levels of the evaluated genes are shown in Table 1. A two-step real-time RT-PCR protocol previously described by Olsvik et al. (2014) was used to quantify the transcriptional levels of the selected genes. Mean normalized expression (MNE) of the target genes was determined using a normalization factor based upon *actb*, *eef1a1*, and *uba52* (*M* < 0.59), as calculated by the *geNorm* software (Vandesompele et al., 2002).

**Table 1:** PCR primers, accession numbers, amplicon sizes, and PCR efficiencies.

Gene symbol	Gene name	Potential marker for	Accession no.	Forward primer	Reverse primer	Amplicon size (bp)	PCR efficiency
*dnmt1*	DNA (cytosine-5-)-methyltransferase 1	DNA methylation	NM_131189	GGGCTACCAGTGCACCTTTG	GATGATAGCTCTGCGTCGAGTC	76	1.91
*dnmt3aa*	DNA (cytosine-5-)-methyltransferase 3A	DNA methylation	NM_001018134	GGCGCCTGTTCTTTGAGTTT	TCACTGACCCCCATTGCAA	112	1.91
*dnmt3b*	DNA (cytosine-5-)-methyltransferase 3B	DNA methylation	NM_001020476	AGGTTTGGAACCTCCCGAAA	TGCGCACAGGTAACAAATGG	115	1.94
*cbs*	Cystathionine-beta-synthase	Transsulfuration	NM_001111232	CTTTGCCCTGGTGGTTCATG	ACCACTCCAAACACCATTTGC	81	2.00
*mgmt*	*O*-6-methylguanine-DNA methyltransferase	DNA repair	NM_001256243	TCCACCCTGTTGTCCTGTCA	GATGTAAGGCAGGCAGAGGAA	117	2.03
*pgrmc1*	Progesterone receptor membrane component 1	Glucose/energy metabolism	NM_001007392	TTTTCACGTCGCCACTGAAC	CTCCTCAACCGGGCCATAGT	104	1.90
*cyp1a1*	Cytochrome P450 family 1 subfamily A member 1	Detoxification	AF210727	GGTGTTGGTTTTCGGTTTGG	GGCATCCCGGTGAACTTTAA	114	1.99
*vtg1*	Vitellogenin 1	Endocrine disruption	NM_001044897	GTCATCAATGAGGATCCAAAGGCCA	GCCTCAGCGATCAGTGCACCAT	209	1.91
*esr1^∗^*	Estrogen receptor 1	Endocrine disruption	NM_152959	AAACACAGCCGGCCCTACAC	GCCAAGAGCTCTCCAACAAC	157	2.12
*esr2a^∗^*	Estrogen receptor 2a	Endocrine disruption	NM_180966	TGATCAGCTGGGCCAAGAAG	GATTAACGGAGCGCCACATC	123	2.00^∗∗^
*ar*	Androgen receptor	Endocrine disruption	NM_001083123	GGATGAGGTCGGAGCAGTTC	GGCTCAATGGCCTCCAGAAT	117	2.03
*cyp19a2*	Cytochrome P450 family 19 subfamily A member 2	Endocrine disruption	AF406756	GAGCGGGCAGGACATAGTGT	GCTTGGGCTCAATCACTCTCA	89	2.10
*fos*	Fos proto-oncogene	Cell proliferation, differentiation and transcription regulation	NM_205569	GGGTATTACCCGCTCAACCA	CAAGTCCGGGCATGAAGAGA	102	2.02
*mapk1*	Mitogen-activated protein kinase 1	Cell proliferation, differentiation and survival	NM_182888	TACATCGGAGGAGGCGCTTA	GCTCAAACGGGCTGATCTTC	94	1.99
*casp3a*	Caspase 3A	Apoptosis	NM_131877	CCCAGATGGTCGTGAAAGGAT	TGAACCATGAGCCGGTCATT	107	2.07
							
*eef1a1*	Eukaryotic translation elongation factor 1 alpha 1	Refgen	AY422992	AGACAACCCCAAGGCTCTCA	CTCATGTCACGCACAGCAAA	126	2.06
*uba52*	Ubiquitin A-52 residue ribosomal protein fusion product 1	Refgen	NM_001037113	CGAGCCTTCTCTCCGTCAGT	TTGTTGGTGTGTCCGCACTT	126	2.08
*actb*	Beta-actin	Refgen	AF057040	CGAGCAGGAGATGGGAACC	CAACGGAAACGCTCATTGC	102	2.08


*^∗^PCR primers obtained from Sawyer et al. (2006). ^∗∗^PCR efficiency set to 2.00.*

### Whole-Genome Bisulfite Sequencing (WGBS)

Bisulfite sequencing was used to identify methylated genomic regions after BPA exposure. Genomic DNA (gDNA) was extracted from five groups of control zebrafish embryos and five groups of embryos exposed to 10 μM BPA for 72 h (from 24 to 96 hpf). The exposure concentration was selected to reflect a sublethal but relatively high dose expected to give behavioral effects using locomotor variant 4 treatment. Each group consisted of pools of 20 embryos. gDNA extraction was conducted with the AllPrep DNA/RNA Mini Kit (Qiagen, Hilden, Germany). After quality validation, and addition of positive control DNA, the gDNA (5 μg) was fragmented to obtain insert size of 200–300 bp with the Covaris S220 Focused-ultrasonicator (Covaris, Woburn, MA, United States). Fragment terminal repair, A-ligation and methylation sequencing adapter ligation were then conducted, using the TruSeq DNA Library Preparation Kit (Illumina, San Diego, CA, United States). Bisulfite treatment was conducted with the EZ DNA Methylation Gold Kit (Zymo Research, Irvine, CA, United States). The ligation products were then purified and size-selected by agarose gel electrophoresis. Bisulfite-treated DNA was subsequently PCR-amplified to enrich both-end adapter fragments. Library concentration was first quantified by Qubit 2.0, and then diluted to 1 ng/μl before checking insert size on the Agilent 2100 Bioanalyzer (Agilent Technologies, Palo Alto CA, United States), followed by more accurate quantification by qPCR (effective concentration of library > 2 nM). Final paired-end sequencing of pooled libraries was done using the HiSeq 4000 platform (Illumina, San Diego, CA, United States).

### WGBS Analysis Pipeline

Quality assessment was completed on raw reads using FastQC 0.11.5 (Andrews, 2016). Adapters and reads under a phred score of 30 were quality trimmed using Trim Galore 0.4.4 (Krueger, 2015). Reads were aligned to the Ensembl *Danio rerio* genome GRCz10 release 87 (Yates et al., 2016) using Bismark 0.18.1 (Krueger and Andrews, 2011). Methylation calling was also completed using Bismark, using the ‘bismark_methylation_extractor’ tool. Three bases were additionally excluded from the 3′ end of each read using the –ignore_3prime and –ignore_3prime_r parameters, to counter methylation bias resulting from the use of unmethylated cytosines in the end-repair step during library preparation.

### Statistics

GraphPad Prism 6.0 software (GraphPad Software, Inc., San Diego, CA, United States) and Statistica 8.0; 2008 (Statsoft Inc., Tulsa, OK, United States) were used for statistical analyses of the transcriptional data. One-way ANOVA with Tukey’s (behavior) or Dunnett’s (transcription) multiple comparisons was used to analyze for possible effects of BPA. In case Bartlett’s test showed that the variances differed, the MNE data were log-transformed before ANOVA analysis. Outliers, as detected by the ROUT method (ROUT Q = 1%), were omitted from the transcriptional dataset. The ROUT method is based on the False Discovery Rate (FDR). With *Q* = 1%, no more than 1% of the identified outliers are false. A significance level of *P* < 0.05 was used for all tests.

Downstream analysis and generation of figures for methylation data were completed using R (R Core Team, 2016). DM sites were identified using methylKit (Akalin et al., 2012). Genomic features were annotated using genomation (Akalin et al., 2015) and GenomicRanges (Lawrence et al., 2013) packages. Promoter regions were defined as 1,000 bp upstream and downstream from transcription start sites. Gene identifiers were annotated using biomaRt (Durinck et al., 2009). All analysis after methylation calling was based on CpG context. DAVID 6.8 was used to identify enriched KEGG pathways (Huang et al., 2009). Global methylation was also represented as a Circos plot (Krzywinski et al., 2009), based on mean hyper and hypomethylation per 10 Mbp tiles.

## Results

### Larval Deformities

**FIGURE 1:**
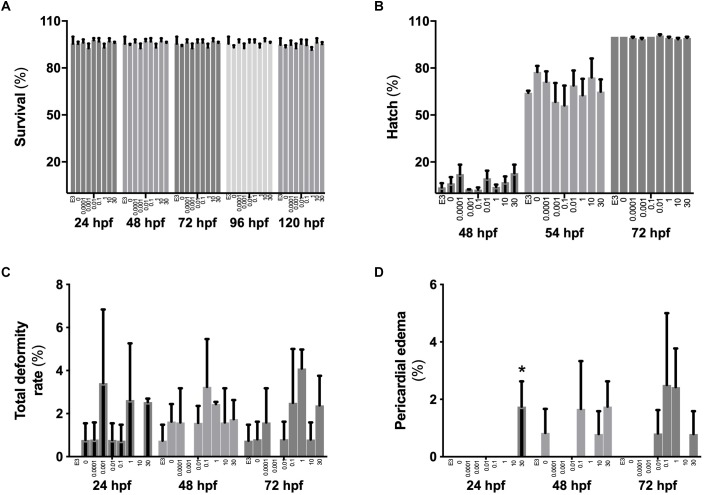
The effects of BPA on zebrafish embryo survival, hatch and rates of deformities. Survival **(A)**, hatch rates **(B)** and rates of total deformities **(C)** or pericardial edema **(D)** in zebrafish embryos when exposed from 2 to 26 hpf to increasing concentrations of BPA. Bars within each time period group represent mean ± SEM, *n* = 3 of E3 controls, DMSO controls, 0.0001, 0.001, 0.01, 0.1, 1, 10, and 30 μM BPA (left to right). Asterisk indicates statistical differences in mean values between treatments (one-way ANOVA, Tukey *post hoc* test, *p* < 0.05).

No differences (*p* > 0.05) were found between controls (E3 media), DMSO vehicle controls or treatments for rates of hatch, survival or total deformities (Figure 1A–C). A small but statistically significant (*p* < 0.05) increase occurred in the rates of pericardial edema in embryos from the highest (30 μM) BPA exposure treatment at 24 hpf (1.8 ± 0.9% vs. 0% in other groups) but not at later stages (Figure 1D).

### Larval Locomotor Activity

**FIGURE 2:**
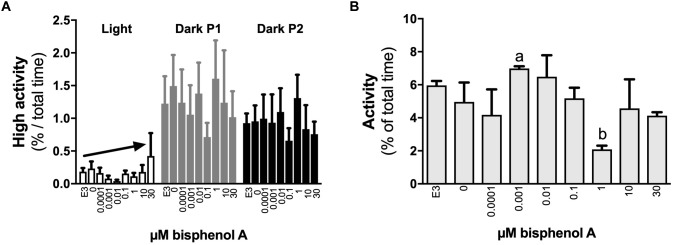
The effects of early developmental exposure to BPA on zebrafish embryo locomotor activity at 4 dpf. The amount of time (% of total) that 4 dpf zebrafish larvae were hyperactive **(A)** or active in general **(B)** when exposed to different light conditions after aqueous exposure to graded levels of BPA (0–30 μM) between 2 and 26 hpf. Light conditions in **(A)** were 5 min full light (open bars) followed by two sequential 5 min periods of darkness (dark P1 and P2, gray or black bars; see locomotor analysis variant 1 in methods). Light conditions in **(B)** were 1 s of dark (dark flash) followed by 9 s of full light, repeated three times (total of 30 s), and the data presented are the average responses over the 30 s period (see locomotor analysis variant 2 in methods). Solid arrows represent linear responses to increasing BPA exposure concentrations (*p* < 0.05, graph **A**, *y* = 0.12 + 0.01*x*, *R*^2^ = 0.17). Letters indicate statistical differences in mean values between treatments (one-way ANOVA, Tukey *post hoc* test, *p* < 0.05). Data are the mean ± SEM, *n* = 3, where each replicate is the average of 8–24 larvae.

**FIGURE 3:**
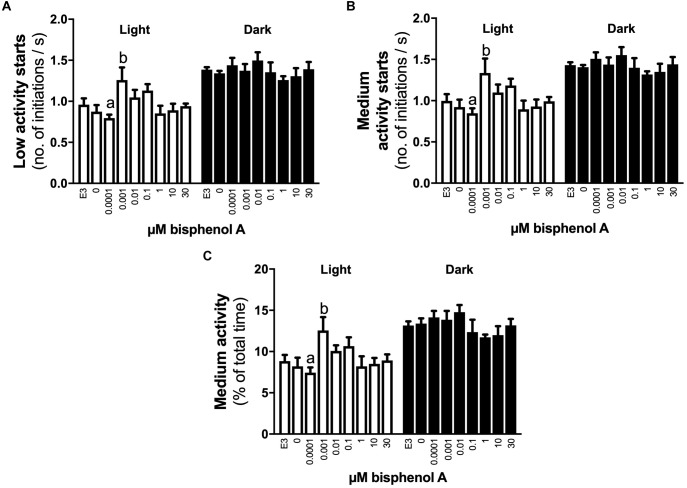
The effects of early developmental exposure to BPA on zebrafish embryo locomotor activity at 5 dpf. The amount of time (% of total) that 5 dpf zebrafish larvae initiated low **(A)** or medium **(B)** levels of activity, and the time medium levels of activity were undertaken **(C)**, under either light (open bars) or dark (black bars) after aqueous exposure to graded levels of BPA (0–30 μM) between 2 and 26 hpf. Letters indicates statistical differences in mean values between treatments (one-way ANOVA, Tukey *post hoc* test, *p* < 0.05). Data are the mean ± SEM, *n* = 3, where each replicate is the average of 8–24 larvae.

**FIGURE 4:**
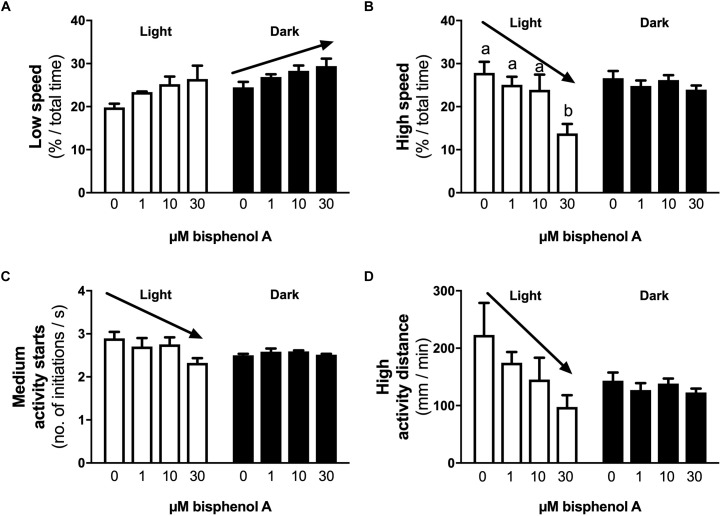
The effects of early developmental exposure to BPA on zebrafish embryo locomotor activity in large wells at 5 dpf (Variant 4, see methods). The amount of time (% of total) that 5 dpf zebrafish larvae moved at low **(A)** or high **(B)** speeds, the number of times zebrafish larvae initiate medium **(C)** levels of activity, and the distance covered when moving at high speeds **(D)**, under either light (open bars) or dark (black bars) after aqueous exposure to graded levels of BPA (0–30 μM) between 2 and 26 hpf. Solid arrows represent linear responses to increasing BPA exposure concentrations (*p* < 0.05, graph **A**, *y* = 26.0 + 0.13*x*, *R*^2^ = 0.36; graph **B**, *y* = 27.1 – 0.43*x*, *R*^2^ = 0.65; graph **C**, *y* = 2.8 – 0.016*x*, *R*^2^ = 0.40; graph **D**, *y* = 196 – 3.5*x*, *R*^2^ = 0.37). Letters indicates statistical differences in mean values between treatments with ANOVA (one-way ANOVA, Tukey *post hoc* test, *p* < 0.05). Data are the mean ± SEM, *n* = 3, where each replicate is the average of 3 larvae.

No differences in locomotor activity were identified at 3 dpf. At 4 dpf, a positive linear response in the percentage of time larvae were hyperactive was identified under light (dark line, linear regression, slope non-zero, *P* = 0.044) but not dark conditions (locomotor analysis variant 1, Figure 2A). When exposed to 1 s dark flashes (locomotor analysis variant 2), larvae exposed to 0.001 μM BPA were active for a larger percent of time over the entire analysis period than those exposed to 1.0 μM BPA (7.0 vs. 2.1% of time, 3.4-fold difference, *p* < 0.05, Figure 2B), but no other differences were observed. No differences between treatments were observed in larvae exposed to a regime of shorter (0.5 s) dark flashes at 4 dpf or 5 dpf (locomotor analysis variant 3, data not shown). At 5 dpf larvae exposed to 0.001 μM BPA initiated low and medium levels of activity more often (1.6-fold) than those exposed to 0.0001 μM BPA under light but not dark conditions (Figure 3A,B). The amount of time that larvae were active at medium activity levels was also higher (1.7-fold) in larvae exposed to 0.001 versus 0.0001 μM BPA (Figure 3C). The locomotor activity of 5 dpf larvae that were developmentally exposed to zero or the three highest concentrations of BPA (1, 10, and 30 μM) were also analyzed in 12 well plates to explore effects under conditions of greater liberty for free movement (locomotor analysis variant 4). Larvae with more room to move demonstrated a positive linear response for time spent moving at low speeds in the dark, but not light conditions (Figure 4A, *P* = 0.039). Negative linear responses to BPA exposure concentrations in the amount of time spent moving at high speeds (Figure 4B, *P* = 0.039). and the number of times medium (Figure 4C, *P* = 0.035) and high (Figure 4D, *P* = 0.030) speed activity was initiated were found under dark but not light conditions.

### BPA-Induced Effects on Gene Expression

**FIGURE 5:**
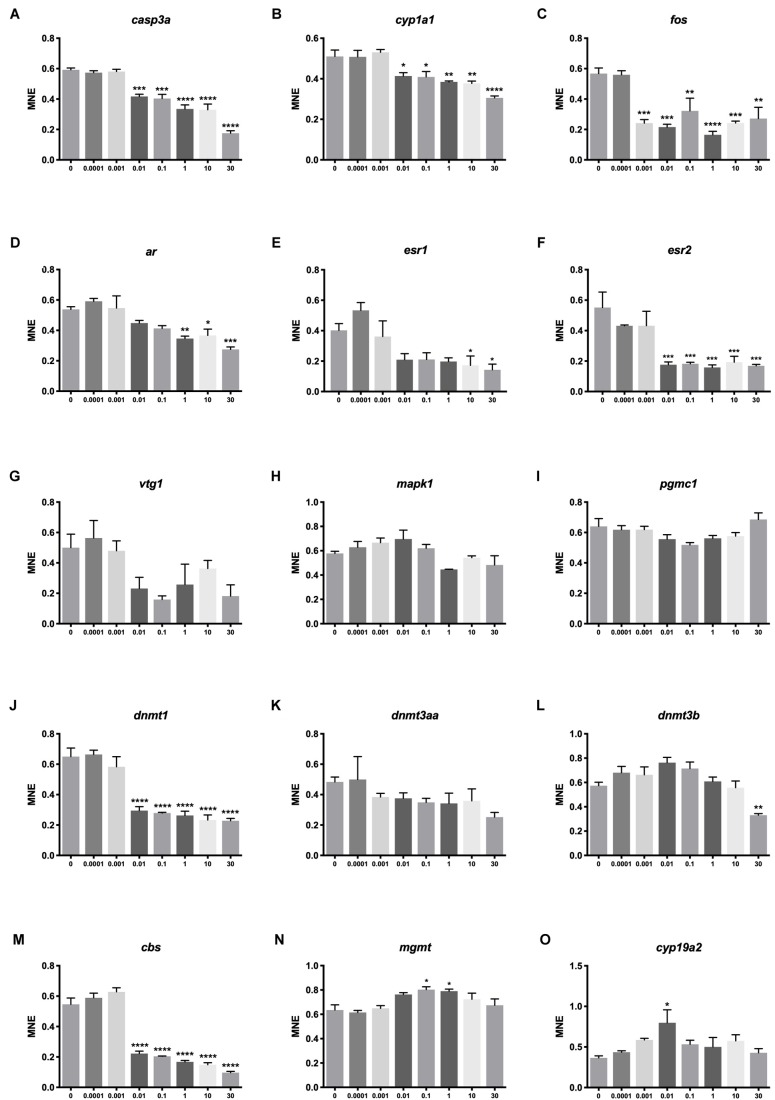
Transcriptional effects of BPA on zebrafish embryos. Concentration-response effects of BPA on selected genes encoding proteins linked to apoptosis **(A,C,N)**, detoxification **(B)**, reproduction **(D–G,O)**, proliferation and differentiation **(H)**, steroidogenesis **(I)**, DNA methylation **(J–L)** and cysteine synthase activity **(M)**. Asterisk indicates statistical differences in mean values between DMSO controls and exposed embryos with ANOVA (one-way ANOVA, Dunnett’s *post hoc* test, ^∗^*p* < 0.05, ^∗∗^*p* < 0.01, ^∗∗∗^*p* < 0.001, ^∗∗∗∗^*p* < 0.0001). Data are the mean ± SEM, *n* = 3, where each replicate is the average of 40 larvae. E3 control not shown.

Significant effects on transcription were seen for 11 out of the 15 examined genes. Several genes were down-regulated at 0.01 μM BPA or higher exposure concentrations. Genes encoding proteins involved in apoptosis (*casp3*, Figure 5A), detoxification (*cyp1a1*, Figure 5B), endocrine disruption (*esr2a*, Figure 5F), DNA methylation (*dnmt1*, Figure 5J) and cysteine synthase activity (*cbs*, Figure 5M) had all lower expression at 0.01 μM BPA or higher concentrations. Responding to even lower concentration, *fos*, encoding a protein also linked to apoptosis, was down-regulated at 0.001 μM BPA (Figure 5C).

The expression of several genes had a non-linear concentration-response to BPA exposure. A pattern of stimulation at lower concentrations followed by inhibition at higher concentrations was observed for *ar*, *esr1*, *mapk1*, *dnmt3b* and *cbs* (Figure 5D,E,H,L,M). Pattern-wise, two genes showed stimulation at medium exposure concentrations (*mgmt*, Figure 5N and *cyp19a2*, Figure 5O), while *pgrmc1* displayed medium concentration inhibition (Figure 5I). At the transcriptional level, BPA therefore seems to induce distinct responses at 0.01 μM or higher concentrations for genes linked to DNA methylation, endocrine disruption, detoxification and cell survival.

### BPA-Induced Effects on DNA Methylation

#### Read Quality and Mapping Rate

An exposure concentration of 10 μM BPA was selected for identification of methylated genomic regions with WGBS based on the behavioral and transcriptional responses. About 50 Gb (range 41–59 Gb) of raw data was generated from each of the five BPA-treated and five control samples (between 140 and 190 million reads per sample). Bisulfite conversion was 99.3% or higher for all samples. A mean of 96.4% of bases had quality scores greater than Q20 and 91.4% scored greater than Q30. Alignment to the GRCz10 zebrafish reference genome yielded a mean mapping rate of 66.4%.

#### Methylation Patterns

**FIGURE 6:**
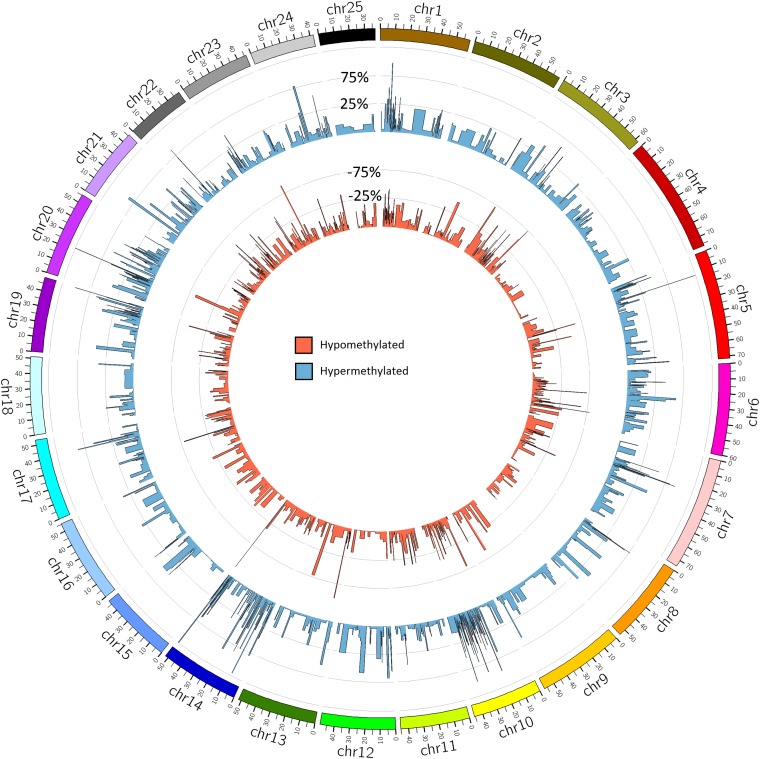
Global methylation patterns in zebrafish embryos exposed to 10 μM BPA from 24 to 96 hpf. Differentially methylated regions in zebrafish embryos exposed to BPA compared to controls. The blue circular bar plot represents percent of hypermethylation and the red circular bar plot shows percent of hypomethylation per region (*Q*-value <0.01 and methylation difference >25%). DM regions in this figure are mean DM per 10 Mbp window.

**FIGURE 7:**
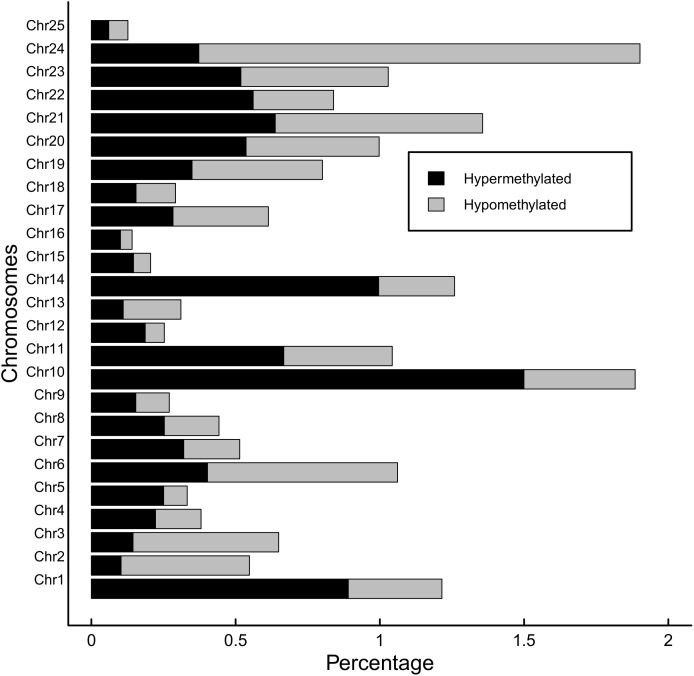
Hypermethylation and hypomethylation per chromosome in zebrafish embryos exposed to 10 μM BPA from 24 to 96 hpf. Percentage of hypermethylated sites per chromosome are shaded black and percentage of hypomethylated regions is gray. Cutoff for differentially methylated sites is *Q*-value <0.01 and methylation difference >25%.

Global methylation was 9.0% (±0.07%) for the BPA treated group and 9.2% (±0.09%) for the control group, though the difference wasn’t significant (*p* = 0.1). Methylation was highest in the CpG context, with 81.7% of CpG sites methylated across all samples. Cytosines in CHG and CHH contexts were each 0.7% methylated. Of these CpG methylated sites, 20,474 sites (18.5% of global CpG methylated sites) were differentially methylated (DM) between BPA treated and control groups, though DM hypermethylated sites (10,981 sites, 9.9% of global CpG methylated sites) were slightly more numerous than hypomethylated (9,493, 8.5%) (Figure 6). Total DM and hyper/hypomethylation was variable between chromosomes (Figure 7). Chromosome 25 and chromosome 16 had the fewest hypermethylated and hypomethylated sites respectively (47 regions each). Chromosomes 10 and 24 had the greatest number of hyper and hypomethylated sites with 1,509 and 1,462 sites respectively.

**FIGURE 8:**
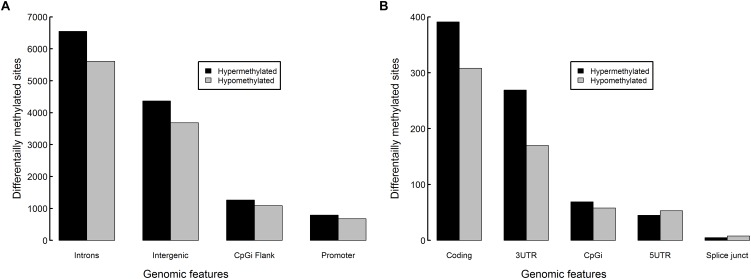
Differentially methylated sites per genomic feature in zebrafish embryos exposed to 10 μM BPA from 24 to 96 hpf. Counts of hypermethylated sites (black) and hypomethylated sites (gray) in **(A)** introns, intergenic regions, CpG island flanks, promoter regions and **(B)** coding, 3′ untranslated regions, CpG islands, 5′ untranslated regions and splice junctions.

Most DM sites fell within introns (12,159 sites) or intergenic regions (8,055 sites) (Figure 8). Promoter regions contained 1,472 DM sites, 1,634 DM sites fell within CpGi flanks, 699 fell within coding regions, 439 fell within 3′ untranslated regions, 64 fell within CpG islands, 98 fell within 5′UTRs and 13 were on splice junctions. Reflecting global methylation, hypermethylated sites were more numerous within introns (53.9% of intron DM sites), intergenic regions (54.2%), CpGi flanks (53.8%), coding regions (55.9%), 3′UTRs (61.3%) and CpG islands (54.7%), though hypomethylation was slightly higher in 5′UTRs (54.1%) and on splice junctions (61.5%). Only 0.6% of CpG islands (CpGi) found within the zebrafish genome were DM. However, 11.3% of the CpGi flanking regions (1,000 bp up and downstream) contained DM sites, which was slightly elevated compared to the 9.1% DM level found over the whole genome.

#### Genes and Metabolic Pathways

Of the 20,474 DM sites, 13,739 of these annotated to zebrafish genes. However, as many genes contain multiple DM sites, the total number of genes containing DM sites was 4,873 (Supplementary Table S1). There were 49 genes containing ≥20 DM sites (Table 2). Of particular significance are the protocadherin 2 gamma subfamily genes, which represent the top seven genes (*pcdh2g1*, *3, 5, 6, 7, 8, 9*), in terms of total number of DM sites per gene and seven out of the nine genes that have more than 50 DM sites. In addition, *pcdh2g12* and *13* both contained 29 DM sites and *pcdh2g17* had 24 DM sites.

**Table 2:** Genes containing ≥20 differentially methylated sites.

Gene symbol	DM sites
*pcdh2g1*	68
*pcdh2g6*	62
*pcdh2g5*	62
*pcdh2g3*	62
*pcdh2g7*	60
*pcdh2g8*	60
*pcdh2g9*	59
*BX005294.2*	59
*si:dkey-33o22.1*	57
*sdk1b*	44
*ntm*	41
*ctdp1*	36
*cntnap2a*	36
*Dlg2*	34
*pcdh1g1*	32
*Klf3*	31
*cux1a*	31
*pcdh2g13*	29
*pcdh2g12*	29
*si:ch211-165d12.4*	28
*cdh4*	27
*csmd3*	27
*magi1b*	26
*msi2a*	26
*ebf1b*	26
*stau2*	25
*dpp6a*	25
*pcdh2aa15*	25
*pcdh1g2*	25
*dmd*	24
*ncoa2*	24
*kcnh5a*	24
*pcdh1a3*	24
*pcdh2g17*	24
*ralgapa2*	23
*nrxn2a*	23
*cadpsb*	23
*pard3b*	23
*dacha*	22
*nudt14*	22
*cadm2a*	21
*uvrag*	21
*pcdh1g3*	21
*vps50*	20
*myrip*	20
*tmem88a*	20
*irrc7*	20
*pcdh2ab6*	20
*pcdh1gb2*	20


Four KEGG pathways were enriched (*p* < 0.01, Benjamini < 0.05) for genes containing DM sites: The phosphatidylinositol signaling system (46 genes), VEGF signaling pathway (32 genes), MAPK signaling pathway (89 genes) and Inositol phosphate metabolism (30 genes) (Table 3). A list of DM genes for each significant KEGG pathway is in Supplementary Table S2. Other metabolic pathways that weren’t significantly enriched, but featured numerous genes with DM sites included Wnt signaling pathway with 50 genes (*p* = 0.005), focal adhesion (*n* = 69, *p* = 0.005), vascular smooth muscle contraction (*n* = 42, *p* = 0.02), adherens junction (*n* = 31, *p* = 0.02), notch signaling pathway (*n* = 20, *p* = 0.02), ErbB signaling pathway (*n* = 32, *p* = 0.03), endocytosis (*n* = 84, *p* = 0.04) and insulin signaling pathway (*n* = 46, *p* = 0.04).

**Table 3:** Enriched KEGG pathways for genes containing differentially methylated cytosines.

KEGG pathway	Count	%	*P*-value	Fold enrichment	Benjamini
Dre04070: Phosphatidylinositol signaling system	46	1.083372586	5.78E-07	2.045716063	8.78E-05
Dre04370: VEGF signaling pathway	32	0.753650495	3.02E-04	1.868899326	0.022686307
Dre04010: MAPK signaling pathway	89	2.096090438	9.44E-04	1.365264965	0.046711852
Dre00562: Inositol phosphate metabolism	30	0.706547339	0.001297272	1.773460107	0.048131455


There were several linkages between gene expression and differential methylation results. Each identified DM site, along with the genome coordinates and genomic feature each site falls within, are listed in Supplementary Table S1. Note that a single DM site may sometimes fall within more than one genomic feature, due to overlapping defined genomic regions per feature. *Dnmt3aa* was hypomethylated at three cytosines, two in introns, and one in the promoter region, but did not show differential gene expression (Figure 5K). Two *dnmt3b* duplicate genes, *dnmt3ba* and *dnmt3bb.1*, contained DM sites: *dnmt3ba* contained two hypomethylated sites, one in the coding, and one in the 3′UTR region and *dnmt3bb.1* contained one hyper and two hypomethylated sites in introns. *Pgrmc1* was hypermethylated in a single promoter region. *Cyp1a1* and *cyp19a2* didn’t contain any DM sites, but four other cytochrome P450 genes did: *cyp2v1*, *cyp2ad2*, *cyp2p10, cyp2p7*, all which contained hypermethylated cytosines within introns. *Esr2a* contained seven hyper and hypomethylated sites, five within in introns and two within promoter regions and *esrra* contained three hypermethylated sites, two in introns and one in the promoter region. *Fos* was not DM but *fosl2*, a zebrafish fos-like antigen, contained one hypomethylated sites in the 3′UTR region. *Mapk1* contained two hypermethylated sites and one hypomethylated site, all within introns. Several other mitogen-activated protein kinases *(and mapk interacting and associated proteins)* were DM, including *mapk11, mapk14a, mapk15, mapk4, mapk7, mapk8ip2, mapk8ip3, mapkap1, mapkbp1 and mapkapk2a*. All of these MAPK genes were DM within introns, with the exception of *mapk7*, which contained a DM site within the promoter region and *mapk4*, with a site within the coding region. Caspase 3a (*casp3a*) contained no DM sites but three other caspases, also involved in apoptosis, were DM: *casp3b, casp6l1, casb8l1*. Casp6l1 was hypomethylated in the promoter region and the other caspase genes were DM in introns.

In the current state of epigenetics research, the functional association between methylation and gene expression is primarily associated with methylation within promoter regions, which has been shown to modify (typically suppress) gene expression. The 1,472 DM sites within promoter regions annotated to 1,147 unique genes (Supplementary Table S3). There were no significantly enriched KEGG pathways, but three pathways contained several genes with promoters containing DM. RNA transport contained 18 genes (*p* = 0.003), lysosome 15 genes (*p* = 0.02) and mRNA surveillance pathway 10 genes (*p* = 0.04).

## Discussion

Associations between contaminant exposure and behavioral effects can rely on altered transcription. Saili et al. (2013) linked hyperactivity observed in zebrafish embryos exposed to 0.1 μM BPA, but not at 80 μM BPA, to altered expression of nervous system genes. More specifically, many genes belonging to the ERK/MAPK signaling and tight junction signaling pathways, which are important in nervous system functioning, were differentially expressed. Of biological functions associated with exposure to 0.1 μM BPA, many described nervous system development or morphology, and 23 genes were included in the “development of central nervous system” function (Saili et al., 2013). Significantly DM sites were observed for several of these genes in this study (*apaf1*, *asic2*, *ift172*, *plxna4*, and *rfx4*), as well as genes closely related to other genes listed in the Saili et al. (2013) study (*celsr1b*, *nlgn1*, *nlgn2a*, *nlgn2b*, *nlgn3a, nlgn4b*, *plxna1a*, *rfxap*, *slc1a1*, *slc1a3b*, and *slc1a7a*). Of these, *apaf1 and nlgn2b* showed DM sites within the promoter region. Though a*sic2* DM sites only fell within introns, the large number of DM sites (21) which were all hypomethylated is too significant a trend to ignore. *Asic2* is functionally relevant, affecting broad neuronal processes and playing a key role in behavioral responses to deleterious stimuli (Price et al., 2014). The exact mechanisms whereby gene body methylation affects gene expression remain unknown, however, this present study provides substantial correlative evidence, further reinforcing the current scientific viewpoint that gene body methylation plays a significant role. Regarding these genes that contained DM sites within promoter regions: a*paf1* is a key transcriptional target that regulates post neural injury apoptosis and neurodegeneration (Fortin et al., 2001) and *nlgn2b* contained two DM sites within its promoter region and six sites within introns, and is a member of the neuroligins gene family, which are involved in the excitation and inhibition of CNS synapses (Rissone et al., 2010). DM sites of these genes observed at 10 μM BPA in this study might thus reflect their altered expression seen in embryos exposed to 0.1 μM (Saili et al., 2013).

A possible link between behavior and development of the central nervous system is also indicated by the DM genes. Intriguingly, the genes with the most DM sites in this study were all protocadherin 2 gamma subfamily genes. Protocadherins, the largest subgroup within the cadherin superfamily, are transmembrane cell–cell adhesion molecules (Chen and Maniatis, 2013). By regulating cell contact formation and stability, cadherins play a crucial role in tissue morphogenesis and homeostasis. Protocadherins are thought to play roles in axon targeting, synaptic development and neuronal survival (El Hajj et al., 2017), although their specific cellular roles in fish remain poorly defined (Biswas et al., 2012). Hypermethylation of protocadherin genes has been shown to result in transcriptional silencing (Waha et al., 2005; Kawaguchi et al., 2008) and of particular significance, methylation of *pcdh* promoters by *dnmt3b* during embryonic stages in mice was demonstrated to modulate the expression of *pcdh* isoforms, affecting neuron development (Toyoda et al., 2014). A possible link between behavior and protocadherin genes has been shown in rats, where the chromosomal region containing the protocadherin-α, -β, and -γ gene families implicated in synaptogenesis showed the highest differential response to maternal care (McGowan et al., 2011). In humans, the protocadherin gamma gene cluster includes 22 genes divided into three subfamilies (The GeneCards Database, 2018). BPA-induced differential methylation of protocadherin genes has previously been reported in humans (Lam et al., 2011; Faulk et al., 2015), and interactions between BPA and protocadherins are frequently reported (Ali et al., 2014; Calhoun et al., 2014; Tait et al., 2015). Differential methylation of protocadherin 2 gamma genes, which are predominantly expressed in the developing nervous system (Sano et al., 1993), might impact neurons, and the behavioral implications in the evolving embryos should be investigated further.

Another possible link between methylation, gene expression and locomotor behavior may be associated with hormone receptor genes involved in sexual maturation. Genes such as *ar*, *pgrmc1* and *esr2a* showed both a reduction in expression at higher BPA concentrations and also contained DM sites, with significantly both *pgrmc1* and *esr2a* containing DM sites within promoter regions. In addition, esr1 showed lower expression and multiple estrogen-related receptor genes contained multiple DM sites. BPA has been demonstrated to both modify methylation and gene expression in estrogen receptor genes and subsequently retard embryo development and maturation of oocytes (Chao et al., 2012; Santangeli et al., 2016).

Global methylation patterns generally followed those observed in other studies. CpG sites are typically highly methylated in vertebrates, around 80%, which correlates with the 81.7% CpG methylation rate detected in this study (Keller et al., 2016). Conversely, CpG islands (CpGi) tend to be mostly unmethylated and are also relatively sparse in the zebrafish genome, for example CpGi were found to be 11-fold greater in stickleback (*Gasterosteus aculeatus*) and tetraodon (*Tetraodon nigroviridis*) than in zebrafish (Han and Zhao, 2008) which may explain why we detected only a small number of DM sites in CpGi regions.

Methylation of promoters can directly regulate gene expression (Farthing et al., 2008; Garcia-Cardona et al., 2014), thereby researchers tend to focus on this, utilizing methods such as targeted SNP arrays or RRBS (Busche et al., 2015). However, these targeted approaches typically ignore methylation in other genomic regions, including gene bodies, where methylation has been shown to also moderate expression via interactions between splice junction and other genomic features (Keller et al., 2016; Yong et al., 2016). Indeed, methylation in gene bodies has been shown to be correlated, and a better indicator of gene expression than promoter methylation in WGBS experiments (Lou et al., 2014). Despite the demonstrated correlation between gene body methylation and expression, the precise function remains unknown, though evidence for direct causal relationships has been found (Yang et al., 2014). Unlike RRBS, which is enriched for promoters and associated CpGi regions, WGBS covers the whole genome and is more reflective of true global methylation patterns, including global CpGi methylation (Dolzhenko and Smith, 2014). We found very high levels of differential methylation in gene bodies (13,739 DM sites in 4,873 genes) which include genes and enriched metabolic pathways that are biologically relevant to BPA exposure. Hypomethylation dominated only in the 5′ untranslated regions and splice junctions, whereas hypermethylation was the predominant response in coding regions, introns, CpG island flanks, promoter regions and 3′UTRs. Further research, covering the whole genome and using methods such as WGBS, is needed to elucidate the implication of contaminant-induced differential methylation of non-promoter genomic regions.

According to the DM genes, four pathways previously linked to BPA exposure were significantly affected by the treatment in zebrafish embryos: Phosphatidylinositol signaling system, VEGF signaling pathway, MAPK signaling pathway and Inositol phosphate metabolism. The phosphatidylinositol (PI) signaling system was the most significantly affected pathway, based on a gene group that included calmodulin (*calm*), CDP-diacylglycerol (*cd*), diacylglycerol kinase (*dgk*), inositol kinases, myotubularin (*mtm*), PI kinases (*pip*), phosphoinositide kinases (*pik*), phospholipase C (*plc*), and protein kinase C (*prk*).

In mice adipose cells, BPA has been shown to act via the PI 3-kinase (PIK3) and AKT kinase pathway, resulting in increased triglyceride accumulation and expression of adipocyte genes (Masuno et al., 2005). Two PIK3 regulatory and catalytic subunit genes, *pik3c3* and *pik3r5*, were DM in this study. PIK3 plays a key role in the insulin signaling pathway, but this pathway was not significantly affected in this study (although many DM genes were found in this pathway). VEGF and MAPK signaling pathways were also predicted to be affected by BPA treatment, based on DM genes, with some overlap between the pathways. The VEGF signaling pathway was predicted to be activated based on differential methylation of genes such as mitogen-activated protein kinases (*mapk*), *pip*, phosphoinositide-3-kinase (*pi3k*), protein kinase C (*prkc*). These genes are also found in phosphatidylinositol signaling system pathway, in addition to protein phosphatase 3 (*ppp3*) and v-akt murine thymoma viral oncogene homologs (*akt3*). Previous mammalian studies have demonstrated that BPA affects mRNA and protein expression of VEGF (Buteau-Lozano et al., 2008; Grasselli et al., 2010; Andersson and Brittebo, 2012; Ptak and Gregoraszczuk, 2015), providing a possible link to altered VEGF signaling by BPA in zebrafish embryos. Based on genes where we found multiple DM sites, several within promoters; such as arrestin (*arr*), calcium channel, voltage-dependent subunits (*cacna*), fibroblast growth factor (*fgf*), *mapk’s*, nuclear factor of activated T-cells (*nfatc*), platelet derived growth factor receptors (*pgdfb*), protein kinase C, protein phosphatase 3 subunits (*ppp3*), RAP1A, member of RAS oncogene family, RAS related proteins, ribosomal protein S6 kinase, TAO kinase, transforming growth factor beta receptor, v-akt murine thymoma viral oncogenes; the MAPK signaling pathway was predicted to be affected. Increased phosphorylation of MAPK1 protein is one of the main effects of BPA in animals, a mechanism also demonstrated in zebrafish (Fitzgerald et al., 2015). BPA has also been shown to affect the expression of *mapk1* in fish (Villeneuve et al., 2012). Lastly, inositol phosphate metabolism was predicted to be affected based on differential methylation of genes such as inositol polyphosphate-5-phosphatases (*inpp5*), inositol-tetrakisphosphate 1-kinases (*itpk*), myotubularin (*mtm*), phosphatidylinositol kinases and phospholipase C (*plc*). As for the other KEGG pathways, a link between BPA exposure and effects on inositol phosphate metabolism has previously been shown in rats (Fernandez et al., 2009). To link the KEGG pathways that have a predicted impact based on differential methylation to altered mRNA expression of the mentioned genes, RNA-seq and WGBS should be performed on the same samples. In this study, the AllPrep DNA/RNA Mini Kit used to isolate DNA failed to produce high-quality RNA which was planned to be used simultaneously for RNA-seq analysis within the current study. However, all four significant KEGG pathways have been linked to the development and function of the nervous system, providing a possible link to the observed behavioral responses. The development of the central nervous system is under tight regulation of numerous signal transduction pathways. For example, the PI, VEGF and MAPK signaling pathways contribute to the regulation of Ca^2+^ in nervous tissue (Fukunaga and Miyamoto, 1998; Lo Vasco, 2012; Mackenzie and Ruhrberg, 2012), offering a potential link the behavioral impacts of BPA in developing embryos. Supplementary Table S4 shows nervous system and developmental diseases and functions affected by BPA exposure in the embryos based on genes with significantly DM promoters or sites. Not only genes with DM promoters, but also genes with DM sites often tend to show a negative correlation with differentially expressed genes (DEGs) (Zhang et al., 2017).

The rates of survival, hatch and deformities demonstrate that the levels of BPA in combination with the exposure period applied in this trial were below those that induced whole organism phenotypic symptoms of toxicity, with the exception of the small increase in pericardial edema observed at the highest BPA (30 μM) concentration. These results are largely in line with previous findings. For example, pericardial and yolk sac edema were identified in embryonic zebrafish at 24 or 120 hpf after exposure to >30 μM BPA from 8 hpf (Saili et al., 2012, 2013). More recent studies have documented changes to pericardial length at lower BPA concentrations (Kinch et al., 2016).

The behavioral responses of larvae to BPA demonstrate that (A) early developmental exposure of BPA can affect larval locomotor activity, (B) light conditions are a factor in response to BPA exposure, (C) BPA can induce hyper or hypoactivity depending on concentration, light conditions and larval age, and (D) over large concentration differences in exposure, BPA appears to induce non-linear responses in locomotor activity. In this trial, there was over five magnitudes of order difference between the lowest (0.0001 μM) and highest (30 μM) BPA exposures. Over these concentration ranges hyperactivity was demonstrated with exposure to 0.001 μM BPA in comparison to embryos exposed to lower or in other instances, higher, BPA concentrations. Kinch et al. (2015) found that exposure to 0.1 μM BPA during different neurogenic periods (16–24 hpf) or (24–36 hpf), but not a pre-neurogenic period (10–16 hpf), resulted in increased locomotor activity at day 5. They showed that BPA induced hyperactivity in embryonic zebrafish at a very low concentration (0.0068 μM), but failed to induce a response at 1.0 μM. In line with our result, these findings demonstrate the non-linear effect of BPA on locomotor activity in embryonic zebrafish. Similarly, embryonic hyperactivity at 5 dpf was observed after neurodevelopmental BPA exposure to 0.01–0.1 μM but not higher (>0.1 μM) or lower (<0.01 μM) BPA concentrations (Saili et al., 2012). Non-linear concentration response patterns are often seen in animals exposed to BPA (Vandenberg, 2014). In adult zebrafish, BPA exposure has been shown to affect swimming at 20 μg/L BPA (0.087 μM), and learning at ≥0.1 μM BPA (Saili et al., 2012; Little and Seebacher, 2015). It thus appears BPA can affect behavioral endpoints in both embryonic and adult zebrafish at very low concentrations.

Based on the behavioral responses and distinct pattern of down-regulation of *casp3a*, *esr2a*, *dnmt1* and *cbs* at 0.01 μM BPA, an exposure concentration of 10 μM BPA was selected for the 24–96 hpf DNA methylation exposure study. The most sensitive of the selected gene markers, *fos*, was down-regulated at 0.001 μM BPA (0.2 μg/L), the lowest concentrations that also induced hyperactivity. Of the genes examined with RT-qPCR, *dnmt3aa*, *dnmt3b*, *pgrmc1*, *esr2a* and *mapk1* had a significantly different number of DM sites and all except *dnmt3b* and *mapk1* contained DM sites within promoter regions. Of these, only *esr2a* and *dnmt3b* responded significantly to BPA exposure according to the transcriptional analyses. Except for *esr2a*, there was therefore no strong linkage between the expression and the number of DM sites or promoters of these well-known markers of BPA exposure. With hyperactivity observed at 0.001 μM BPA, and significantly altered expression of some of the selected genes from 0.001 to 0.01 μM BPA, a lower exposure concentration for the DNA methylation exposure experiment might have provided a stronger link between the studied endpoints.

Although significant only for *esr2a* and *ar*, BPA at 0.01 μM or higher concentrations appears to down-regulate genes related to sex hormone metabolism. Up-regulation of estrogen-related genes is one of the best-documented effects of endocrine disrupting chemicals such as BPA in fish (Santos et al., 2014; Sun et al., 2014; Zhang et al., 2014; Mathieu-Denoncourt et al., 2015; Qiu et al., 2016). In line with this, an upstream regulator analysis included in the IPA core analysis, comparing the genes with DM promoters (with IPA mapping 1,426 out of a total of 1,866 genes), predicted beta-estradiol, followed by MYCN and LIMS, as the top upstream regulator based on 81 target molecules (Supplementary Table S5). Beta estradiol (followed by ESR1 and topotecan) was also the top predicted upstream regulator using all genes with DM sites, based on 294 target molecules (IPA mapped 10,821 out of 13,929 DM site genes) (Supplementary Table S5). Of other estrogen-related genes examined in this study, *vtg1* was not significantly affected by BPA exposure (Figure 5G), however, its response pattern resembled those of *esr1* and *esr2a*. In various fish species, BPA-induced *vtg1* expression has been observed at concentrations below 53 μM (Vandenberg et al., 2013). This study thus further demonstrates BPA-induced dysregulation of the sex hormone steroid axis in zebrafish.

In line with earlier zebrafish studies (Calhoun et al., 2014; Chen et al., 2015; Laing et al., 2016), BPA at >0.01 μM exposure down-regulated *dnmt1*. Reduced *dnmt1* transcription was, however, not associated with reduced global DNA methylation in the current study. There was a slight trend toward reduced global DNA methylation (9.0% for BPA treated vs. 9.2% global methylation in control group, *p* = 0.1) in embryos exposed to 10 μM BPA for 72 h. In line with our result, Bouwmeester et al. (2016) examined global DNA methylation in zebrafish embryos exposed to 4.9–27.7 μM BPA for various lengths and observed no effects. Other chemicals also failed to induce changes at the global DNA methylation level. The authors speculated that absence of effects on the global scale might indicate that responses are too small in whole-embryos, even with numerous site-specific methylation modifications (Bouwmeester et al., 2016). These findings suggest that DNA modifications at the global scale might not be a sensitive marker of epigenotoxicity in fish whole-embryo samples.

## Conclusion

Significant effects on transcriptional endpoints were seen in zebrafish embryos exposed to >0.001 μM BPA 2–26 hpf, and in behavioral endpoints in embryos exposed to 1–30 μM BPA 24–96 hpf. In embryos exposed to 10 μM between 24 and 96 hpf, promoter regions showed higher hypomethylation than hypermethylation. Most DM genes, however, occurred in gene bodies and these were hypermethylated. The study further identified protocadherin 2 gamma subfamily genes, plus *apaf1*, *asic2*, *ift172*, *plxna4*, and *rfx4*, as potential candidates linking DNA methylation and behavioral endpoints in developing fish larvae. According to the DM genes, phosphatidylinositol-, VEGF-, and MAPK signaling pathways and inositol phosphate metabolism were significantly affected by the treatment in zebrafish embryos.

## Author Contributions

PO conceived the project, with all co-authors providing advise at various stages of the project, analyzed the transcriptional data, and interpreted all data. PO, SP, and SE designed the experiments, while SP, KS, and SE conducted the experiments. SP analyzed and interpreted the behavioral data. PW developed the WGBS bioinformatics pipeline and analyzed the DNA methylation data. PO, SP, and PW wrote the manuscript and KS, MA, and SE edited the manuscript. All authors read and approved the final manuscript.

## Conflict of Interest Statement

The authors declare that the research was conducted in the absence of any commercial or financial relationships that could be construed as a potential conflict of interest.

## Supplementary Material

Table S1 Annotated table of DM sites found, including genomic features DM sites fell within, methylation difference (%), gene annotation (Ensembl gene ID, Entrez gene ID, gene symbol and description), genomic coordinates (chromosome, start, end, strand) and *p*/*q* significance values.

Table S2 DM genes for each significant KEGG pathway.

Table S3 DM sites within promoter regions.

Table S4 IPA predicted top upstream regulators based on genes with DM sites.

Table S5 IPA predicted top upstream regulators based on genes with DM promoters.
