# Corrigendum: Associations Between Behavioral Effects of Bisphenol A and DNA Methylation in Zebrafish Embryos

**DOI:** 10.3389/fgene.2020.00107

**Published:** 2020-02-12

**Authors:** Pål A. Olsvik, Paul Whatmore, Sam J. Penglase, Kaja H. Skjærven, Marc Anglès d’Auriac, Ståle Ellingsen

**Affiliations:** ^1^Institute of Marine Research, Bergen, Norway; ^2^Norwegian Institute for Water Research, Oslo, Norway

**Keywords:** zebrafish embryos, bisphenol A, behavior, gene expression, DNA methylation, epigenetics

In the original article, there was a mistake in **Table 1** as published. Due to a copy-paste error, the accession numbers, PCR primers, and amplicon sizes given for two of the RT-qPCR assays, *mapk1* and *casp3a*, were wrong. Also, the accession number for *dnmt1* was incorrect. The corrected **Table 1** appears below. The authors apologize for this error and state that this does not change the scientific conclusions of the article in any way. The original article has been updated.

**Table 1:** PCR primers, accession numbers, amplicon sizes, and PCR efficiencies.

Gene symbol	Gene name	Potential marker for	Accession no.	Forward primer	Reverse primer	Amplicon size (bp)	PCR efficiency
*dnmt1*	DNA (cytosine-5-)- methyltransferase 1	DNA methylation	NM_131189	GGGCTACCAGTGCACCTTTG	GATGATAGCTCTGCGTCGAGTC	76	1.91
*dnmt3aa*	DNA (cytosine-5-)- methyltransferase 3A	DNA methylation	NM_001018134	GGCGCCTGTTCTTTGAGTTT	TCACTGACCCCCATTGCAA	112	1.91
*dnmt3b*	DNA (cytosine-5-)- methyltransferase 3B	DNA methylation	NM_001020476	AGGTTTGGAACCTCCCGAAA	TGCGCACAGGTAACAAATGG	115	1.94
*cbs*	Cystathionine-beta-synthase	Transsulfuration	NM_001111232	CTTTGCCCTGGTGGTTCATG	ACCACTCCAAACACCATTTGC	81	2.00
*mgmt*	O-6-methylguanine-DNA methyltransferase	DNA repair	NM_001256243	TCCACCCTGTTGTCCTGTCA	GATGTAAGGCAGGCAGAGGAA	117	2.03
*pgrmc1*	Progesterone receptor membrane component 1	Glucose/Energy metabolism	NM_001007392	TTTTCACGTCGCCACTGAAC	CTCCTCAACCGGGCCATAGT	104	1.90
*cyp1a1*	Cytochrome P450 family 1 subfamily A member 1	Detoxification	AF210727	GGTGTTGGTTTTCGGTTTGG	GGCATCCCGGTGAACTTTAA	114	1.99
*vtg1*	Vitellogenin 1	Endocrine disruption	NM_001044897	GTCATCAATGAGGATCCAAAGGCCA	GCCTCAGCGATCAGTGCACCAT	209	1.91
esr1*	Estrogen receptor 1	Endocrine disruption	NM_152959	AAACACAGCCGGCCCTACAC	GCCAAGAGCTCTCCAACAAC	157	2.12
esr2a*	Estrogen receptor 2a	Endocrine disruption	NM_180966	TGATCAGCTGGGCCAAGAAG	GATTAACGGAGCGCCACATC	123	2.00**
*ar*	Androgen receptor	Endocrine disruption	NM_001083123	GGATGAGGTCGGAGCAGTTC	GGCTCAATGGCCTCCAGAAT	117	2.03
*cyp19a2*	Cytochrome P450 family 19 subfamily A member 2	Endocrine disruption	AF406756	GAGCGGGCAGGACATAGTGT	GCTTGGGCTCAATCACTCTCA	89	2.10
*fos*	Fos proto-oncogene	Cell proliferation, differentiation and transcription regulation	NM_205569	GGGTATTACCCGCTCAACCA	CAAGTCCGGGCATGAAGAGA	102	2.02
*mapk1*	Mitogen-activated protein kinase 1	Cell proliferation, differentiation and survival	NM_182888	TACATCGGAGGAGGCGCTTA	GCTCAAACGGGCTGATCTTC	94	1.99
*casp3a*	Caspase 3A	Apoptosis	NM_131877	CCCAGATGGTCGTGAAAGGAT	TGAACCATGAGCCGGTCATT	107	2.07
*eef1a1*	Eukaryotic translation elongation factor 1 alpha 1	Refgen	AY422992	AGACAACCCCAAGGCTCTCA	CTCATGTCACGCACAGCAAA	126	2.06
*uba52*	Ubiquitin A-52 residue ribosomal protein fusion product 1	Refgen	NM_001037113	CGAGCCTTCTCTCCGTCAGT	TTGTTGGTGTGTCCGCACTT	126	2.08
*actb*	Beta-actin	Refgen	AF057040	CGAGCAGGAGATGGGAACC	CAACGGAAACGCTCATTGC	102	2.08

*PCR primers obtained from Sawyer et al. [93]. **PCR efficiency set to 2.00.

